# Molecular Characterization of HER2-Low Invasive Breast Carcinoma by Quantitative RT-PCR Using Oncotype DX Assay

**DOI:** 10.1093/oncolo/oyad249

**Published:** 2023-09-01

**Authors:** Hao-Kuen Lin, Thuy Can, Adriana Kahn, Cynthia A Flannery, Jess Hoag, Alekhya Akkunuri, Helen Bailey, Rick Baehner, Lajos Pusztai, Mariya Rozenblit

**Affiliations:** Department of Medical Oncology, Yale School of Medicine, Yale Cancer Center, New Haven, CT, USA; Exact Sciences Corporation, Redwood City, CA, USA; Department of Medical Oncology, Yale School of Medicine, Yale Cancer Center, New Haven, CT, USA; Exact Sciences Corporation, Redwood City, CA, USA; Exact Sciences Corporation, Redwood City, CA, USA; Exact Sciences Corporation, Redwood City, CA, USA; Exact Sciences Corporation, Redwood City, CA, USA; Exact Sciences Corporation, Redwood City, CA, USA; Department of Medical Oncology, Yale School of Medicine, Yale Cancer Center, New Haven, CT, USA; Department of Medical Oncology, Yale School of Medicine, Yale Cancer Center, New Haven, CT, USA

**Keywords:** HER2-low, Oncotype DX assay, HER2 RT-PCR, recurrence score, breast cancer

## Abstract

**Background:**

HER2 immunohistochemistry (IHC) reproducibility is suboptimal for HER-low cases (IHC 1+ or 2+).

**Methods:**

The Yale cohort included 214 stages I-II estrogen receptor positive breast cancers with IHC scores 0, 1+, and 2+ and routine Oncotype DX Recurrence Score (RS) results. The Exact Sciences (ES) cohort included 9 57 624 patients who had an Oncotype DX RS assay that assigns HER2-negative, equivocal, or positive status based on HER2 mRNA levels.

**Results:**

HER2 mRNA levels varied across IHC categories but with increasing medians of 9.10 (*n* = 89), 9.20 (*n* = 71), and 9.45 (*n* = 54) in IHC 0, 1+, and 2+, respectively. 22.4% of HER2-low (1+/2+) cancer had RS > 25. Over 98% of HER-low cancers were HER2-negative by Oncotype DX assignment.

**Conclusions:**

Cancers with higher mRNA levels exist within IHC 0 and low categories, most of the HER2-low patients by IHC have low RS indicating no benefit from current adjuvant chemotherapies.

## Teaser

This article assesses the relationship between HER2 mRNA levels by Oncotype DX assay and HER2 immunohistochemistry categories and examines recurrence score distribution across the HER2 categories by HER mRNA levels.

## Introduction

The DESTINY-Breast04 trial demonstrated efficacy of trastuzumab deruxtecan (T-DXd), an anti-HER2 (human epidermal growth factor receptor 2) antibody-drug conjugate, in HER2-low metastatic breast cancer.^1^ HER2-low was defined as immunohistochemistry (IHC) 1+ or 2+ with no amplification by in situ hybridization (FISH). However, the ­reproducibility of HER2 IHC is poor, which may lead to misclassification for T-DXd treatment.^2^ Recent studies have investigated clinicopathological characteristics of HER2-low group with inconsistent results,^3-8^ partly explained by inconsistencies in HER2 IHC assignments.

In the DESTINY-Breast04 trial, 90% of the participants were ER+.^1^ Multiple studies also showed that about half of all ER+ cancers are HER-low with IHC.^3-8^ For early-stage ER + breast cancers, the Oncotype DX Recurrence Score (RS) assay is used to guide adjuvant chemotherapy.^9^ The Oncotype DX also provides standardized HER2 mRNA levels by RT-PCR. There is high concordance between HER2 protein and HER2 mRNA expression levels.^10^ HER2 mRNA level or RS distributions across the HER2-low and HER2-0 groups have not yet been reported. The main goal of this study is to assess the relationship between HER2 mRNA levels by Oncotype DX assay and HER2 IHC categories. We also examined RS distribution across the HER2 categories by HER mRNA levels.

## Materials and Methods

This study was approved by the Yale and WIRB-Copernicus Group Institutional Review Boards and includes 2 patient cohorts. The Yale cohort included 214 ER + stage I-II cancers with HER2 IHC 0, 1+, and 2+, who were HER2 negative by FISH and had RS results were obtained through routine care between 2012 and 2017. Patients with high-RS results are defined as scores > 25.^9^ Histologic grade and Ki-67 percent positivity determined in the context of routine pathologic evaluation were retrieved from the medical records. High Ki-67 index is defined as ≥20%. The Exact Sciences (ES) cohort included 957 624 patients who were tested between 2005 and 2021; HER2 mRNA level and proliferation group scores were also examined. IHC were not available. The Oncotype DX assay assigns HER2 negative (HER2 mRNA < 10.7), HER2 equivoical (≥10.7 to <11.5), and HER2-positive status (≥11.5) based on quantitative RT-PCR measures.

The Chi-squared test was used to compare categorical variables across HER2 IHC categories, and Fisher’s exact test was used to compare Ki-67 percent positivity and HER2 groups by RT-PCR. HER2 mRNA level, RS, and Ki-67 percent positivity were compared using the 2-sided Mann-Whitney *U* test. Analyses and figure generation were performed in R version 3.6.1, Python 3.9, and using SAS v9.4 (SAS Institute, Cary NC).

## Results

The clinicopathologic and molecular characteristics of the Yale cohort are presented in Table 1. 42%, 33%, and 25% of cases were IHC 0, 1+, and 2+, respectively. There was no difference in age and tumor grade by IHC category (Table 1). Among the HER2 IHC 2+ cases, there was one case that was HER2 positive by OncotypeDx.

**Table 1.** Clinicopathologic and molecular characteristics by HER2 IHC group.

Clinical values	HER2 IHC 0 (*n* = 89) No. (%)	HER2 IHC 1+ (*n* = 71) No. (%)	HER2 IHC 2+ (*n* = 54) No. (%)	Chi-square test (Fisher’s Exact test) *P*-value
Age (years)				
<50	9 (10)	12 (17)	7 (13)	.45
≥50	80 (90)	59 (83)	47 (87)	
Tumor grade				
1	31 (35)	31 (44)	12 (22)	.16
2	47 (53)	32 (45)	32 (59)	
3	11 (12)	8 (11)	10 (19)	
Ki-67 percent positivity^a^				
Low (<20%)	36 (97)	31 (89)	12 (75)	.03
High (≥20%)	1 (3)	4 (11)	4 (25)	
HER2 RT-PCR				
Positive (≥11.5)	0	0	1 (2)	.06
Equivocal (10.7-11.5)	0	0	1 (2)	
Negative (<10.5)	89 (100)	71 (100)	52 (96)	
Recurrence score				
Low (≤25)	77 (87)	61 (86)	36 (67)	.006
High (>25)	12 (13)	10 (14)	18 (33)	

^a^88 out of 214 patients had Ki-67 IHC performed.

**Figure 1.**
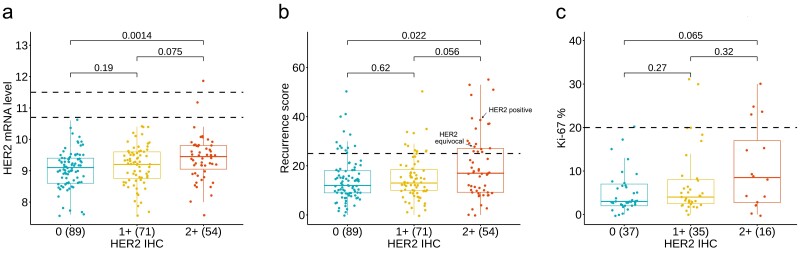
Distribution of different molecular and pathological features across 3 HER2 IHC categories. Boxplots of (**A**) HER2 mRNA level, (**B**) recurrence score, and (**C**) Ki-67 percent positivity across HER2 IHC 0, 1+, and 2+ invasive breast cancers. The number of patients is shown in brackets. Mann-Whitney *U* test was used to test difference in median across groups. The black dotted lines in (A) represent HER2 mRNA level cutoffs for negative, equivocal, and positive (10.7 and 11.5), (B) represent high RS threshold (25), and (C) represent high Ki-67 index threshold (20). In (B), the samples that are HER2-positive or equivocal by mRNA are labeled.

HER2 mRNA levels increased across increasing IHC categories with medians 9.10, 9.20, 9.45, respectively. In group-wise comparisons, only IHC 0 compared with IHC 2+ reached statistical significance (Mann-Whitney *U* test, *P* = .001, Fig. 1). There was substantial variation in HER2 mRNA level in all IHC groups.

RS results were also significantly higher in IHC 2+ vs. 0 cases (medians 17.0 vs. 12.0, Mann-Whitney *P* = .02, Fig. 1). Among IHC-low (1+/2+) cases 22.4% had high RS (Table 1).

The frequency of high Ki-67 expression was also significantly different in the 3 IHC cohorts, 3% in IHC 0, 11% in IHC 1+, and 25% in IHC 2+ (*P* = .03). We further tested the association between HER2 mRNA level and HER2 IHC categories after removing HER2-positive or -equivocal samples by OncotypeDX, IHC 2+ cancers continued to show higher mRNA levels compared to IHC 0 (Supplementary Fig. S1). Similarly, IHC 2+ had higher RS results compared to IHC 0 (Supplementary Fig. S1 and Supplementary Table S1).

In the ES cohort (Supplementary Table S2), 0.8% of samples were HER2 positive, 1.2% were equivocal, and 98% were negative by RT-PCR using the predefined thresholds. There was a wide range of RS within all 3 categories. Of the HER2-positive cases by RT-PCR, 94.7% had high RS. Of the HER2-equivocal cases, 39.1% had high RS results, and in HER2-negative cases, that correspond to the majority of IHC-low cases based on findings in the Yale cohort, 15.5% had high RS results. There was a weak correlation between HER2 mRNA level and both RS results (Supplementary Fig. S2, Spearman correlation = −0.27) and the proliferation group score (Supplementary Fig. S3, Spearman correlation = −0.05).

## Discussion

Results from the DESTINY-Breast04 and DAISY trials generated interest in accurate identification of HER2-low breast cancers.^1,11^ The DAISY trial included a cohort of metastatic breast cancers that were HER2-negative *n* = 38, IHC 0/FISH negative and were treated with T-DXd, 23% of these patients experienced tumor response.^11^ This unexpected result is attributed to false negative IHC staining and demonstrates the limitation of IHC to select patients for T-DXd therapy. Quantification of HER2 mRNA is standardized and is ­highly reproducible by using the OncotypeDx assay. We examined HER2 mRNA expression levels and RS result distribution among HER2 IHC 0, 1+, and 2+ cases using data from early-stage cancers. While, HER2 mRNA levels increased as HER2 expression by IHC increased, there were large within group variations in HER2 mRNA levels indicating heterogeneous HER2 expression within IHC categories. This raises the possibility that mRNA-based threshold could be defined that might predict treatment response to T-DXd better than IHC results. Defining thresholds is only possible if treatment response is available. We also noted that the majority (87.6%) of IHC HER2-low cancers had low RS results and, therefore, have no predicted benefit from adjuvant chemotherapy. Clinical trials are needed to determine if these patients, when clinically high risk, might benefit from novel ADCs targeting HER-low cancers.

This study used data generated from early-stage breast cancers rather than metastatic cancer biopsies. The HER2 mRNA quantification method (RT-PCR) is robust to variable tissue composition and should yield reliable results in various types of biopsies, and HER2 expression tends to remain stable during the course of the disease.^12,13^ However, discordant HER2 expression results by IHC between primary tumor and metastatic biopsies can be seen, and Oncotype DX and its components have not been validated for metastatic biopsies.

## Conclusion

HER2 IHC 0 and IHC HER2-low cancers have a broad and overlapping range of HER2 mRNA levels. This raise the possibility that T-DXd sensitive subpopulations exist across all these groups and an mRNA-based eligibility threshold could be constructed if treatment response data were available. Almost all IHC HER2-low breast cancers have low RS results, indicating no benefit from current adjuvant chemotherapies.

## Supplementary Material

oyad249_suppl_Supplementary_Figure_S1

oyad249_suppl_Supplementary_Figure_S2

oyad249_suppl_Supplementary_Figure_S3

oyad249_suppl_Supplementary_Tables

## Data Availability

The data underlying this article will be shared on reasonable request to the corresponding author.
