# Supplementary figures and images for: Molecular Characterization of HER2-Low Invasive Breast Carcinoma by Quantitative RT-PCR Using Oncotype DX Assay
Source: Oncologist. 2023 Sep 1;28(10):e973–6. doi: 10.1093/oncolo/oyad249 (PMC10546821; doi:10.1093/oncolo/oyad249)

**a**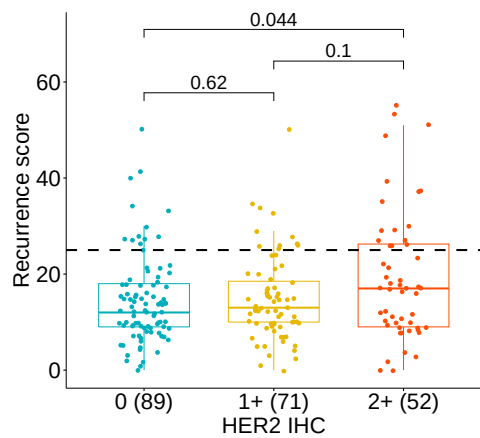**b**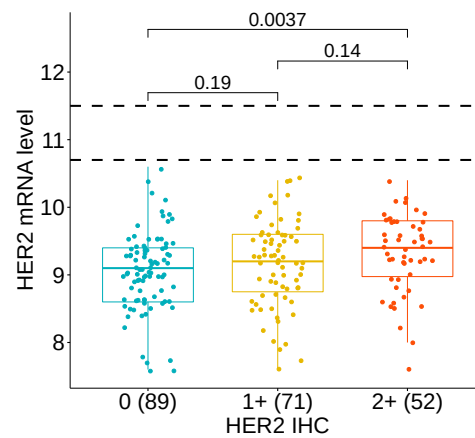

Supplement: oyad249_suppl_Supplementary_Figure_S1 [file oyad249_suppl_supplementary_figure_s1.pdf]

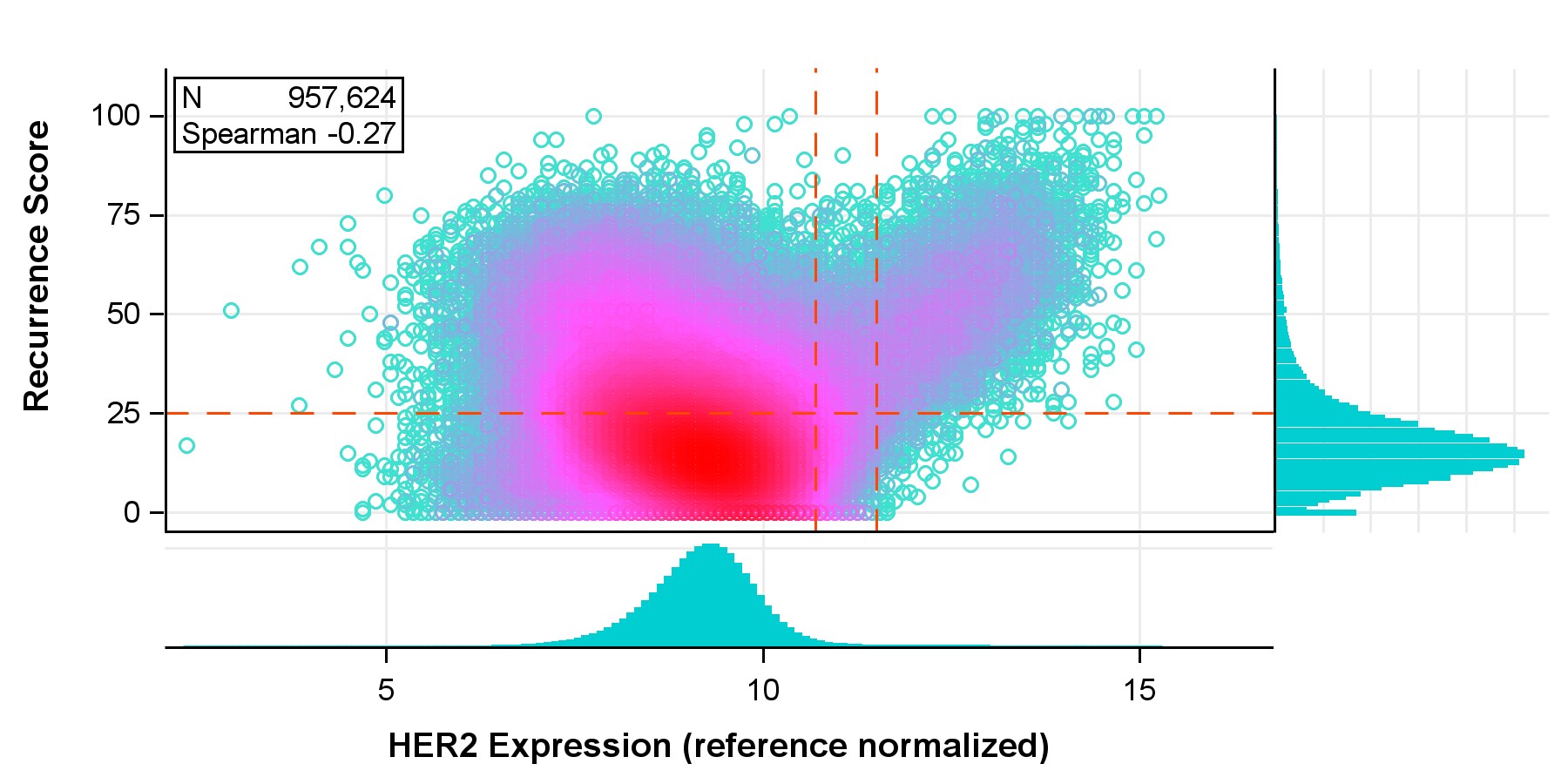

Supplement: oyad249_suppl_Supplementary_Figure_S2 [file oyad249_suppl_supplementary_figure_s2.jpeg]

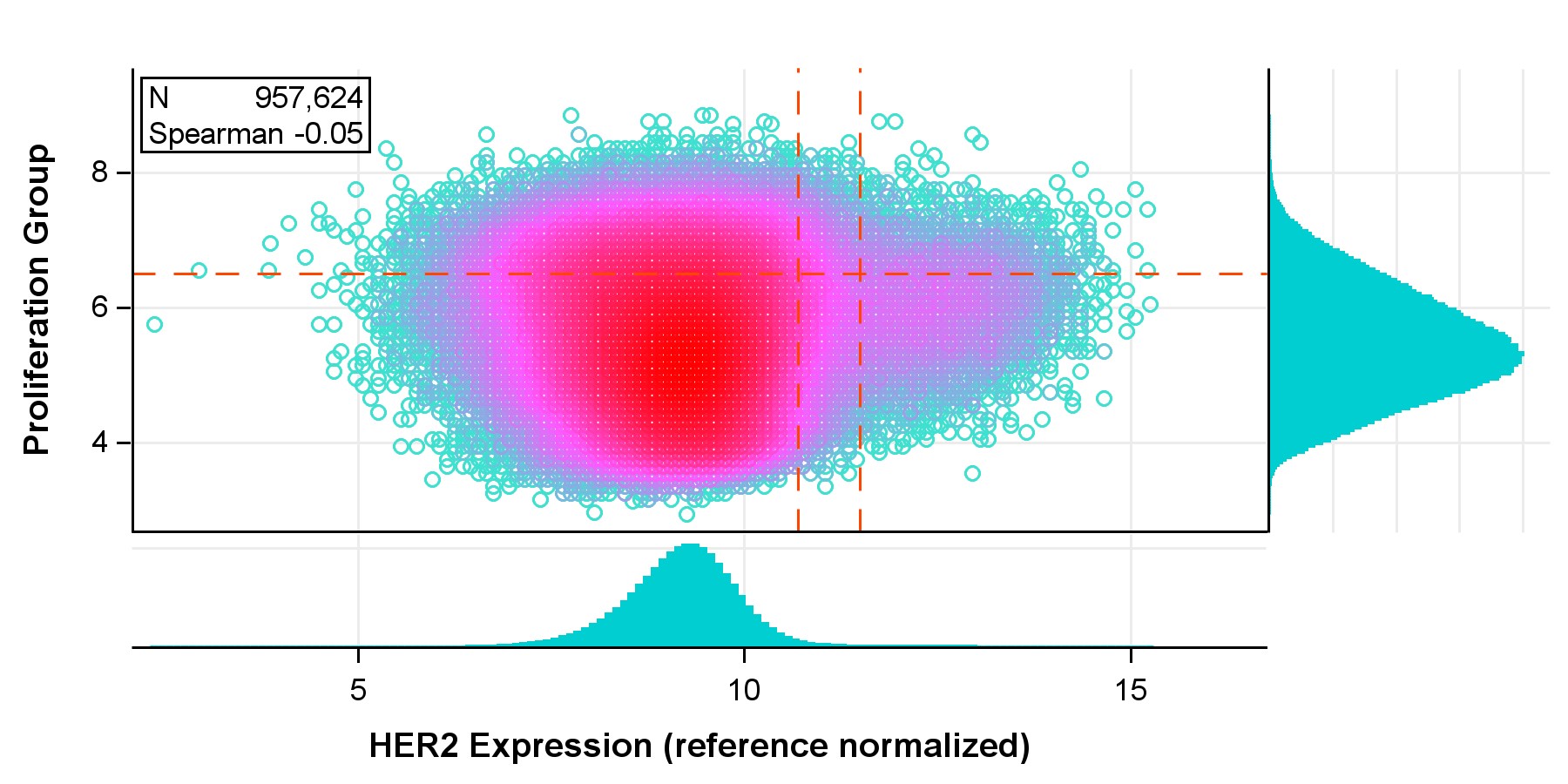

Supplement: oyad249_suppl_Supplementary_Figure_S3 [file oyad249_suppl_supplementary_figure_s3.jpeg]
