# Supplementary material for: Molecular Characterization of HER2-Low Invasive Breast Carcinoma by Quantitative RT-PCR Using Oncotype DX Assay
Source: Oncologist. 2023 Sep 1;28(10):e973–6. doi: 10.1093/oncolo/oyad249 (PMC10546821; doi:10.1093/oncolo/oyad249)
Supplement: oyad249_suppl_Supplementary_Tables [file oyad249_suppl_supplementary_tables.docx]

**Supplementary Table S1. RS by HER2 IHC categories**

| **Clinical values** | **HER2 IHC 0**  **(n = 89)**  **No. (%)** | **HER2 IHC 1+**  **(n = 71)**  **No. (%)** | **HER2 IHC 2+**  **(n = 52)**  **No. (%)** | **Chi-square test *p*-value** |
| --- | --- | --- | --- | --- |
| **Recurrence Score** |  |  |  |  |
| Low (≤25) | 77 (87) | 61 (86) | 36 (70) | 0.02 |
| High (>25) | 12 (13) | 10 (14) | 16 (30) |  |

**Supplementary Table S2. Quantitative HER2 gene expression across HER2 IHC groups in the Exact Sciences cohort**

| **HER2 RT-PCR** | **Number of patients** | **Low (RS ≤25)** | **High (RS >25)** |
| --- | --- | --- | --- |
| **Positive (≥11.5)** | 7,184 | 381 (5.3%) | 6,803 (94.7%) |
| **Equivocal**  **(≥10.7 and <11.5)** | 11,528 | 7,022 (60.9%) | 4,506 (39.1%) |
| **Negative (<10.7)** | 938,912 | 793,803 (84.5%) | 145,109 (15.5%) |

**Supplementary Figure 1. Distribution of different molecular and pathological features across three HER2 IHC categories removing HER2 equivocal and positive samples by mRNA.** (a) HER2 mRNA level, and (b) Recurrence Score across HER2 IHC 0, 1+ and 2+ invasive breast cancers. The number of patients is shown in brackets. Mann-Whitney U test was used to test difference in median across groups. The black dotted lines in (a) represent HER2 mRNA level cutoffs for negative, equivocal and positive (10.7 and 11.5), and (b) represent high RS threshold (25).

**Supplementary Figure 2. Distribution of quantitative HER2 gene expression and RS result**

Scatter plot of HER2 expression and Recurrence Score result from Exact Sciences cohort. Circles are colored by the density. The vertical red dotted lines represent the cutoffs for HER2 positive, equivocal, and negative (11.5 and 10.7). The horizontal red dotted line represents the threshold for Recurrence Score > 25.

**Supplementary Figure 3. Distribution of quantitative HER2 gene expression and proliferation group score**

Scatter plot of HER2 expression and proliferation score from Exact Sciences cohort. Circles are colored by the density. The vertical red dotted lines represent the cutoffs for HER2 negative, equivocal and positive (10.7 and 11.5). The horizontal red dotted line represents the threshold for proliferation group score (6.5).
